# Investigation on the Mechanism of PAL (100) Surface Modified by APTES

**DOI:** 10.3390/molecules28145417

**Published:** 2023-07-14

**Authors:** Weimin Jia, Bomiao Qi, Yanbin Wang, Zhibin Lu, Jiqian Wang, Qiong Su, Jingyan Nian, Junxi Liang

**Affiliations:** 1 School of Chemical Engineering, Northwest Minzu University, Lanzhou 730030, China; jweiminsw@126.com (W.J.); qi2640642157@163.com (B.Q.); ybwang@126.com (Y.W.); 2 State Key Lab Solid Lubricat, Lanzhou Institute of Chemical Physics, Lanzhou 730000, China; zblu@licp.cas.cn (Z.L.); sweetnjy@licp.cas.cn (J.N.); 3 Nanjing Research Institute of Electronics, Nanjing 210039, China; wjqisy@163.com

**Keywords:** palygorskite (PAL), 3-aminopropyltriethoxysilane (APTES), density functional theory, binding energy, mechanism

## Abstract

The interfacial mechanism has always been a concern for 3-aminopropyltriethoxysilane (APTES)-grafted palygorskite (PAL). In this research, the mechanism of graft modification for grafting of APTES to the surface of PAL (100) was studied using density functional theory (DFT) calculation. The results illustrated that different grafting states of the APTES influence the inter- and intramolecular interactions between APTES/PAL (100), which are reflected in the electronic structures. For single-, double-, and three-toothed state APTES-PAL (100), the charge transfer rates from the PAL (100) surface to APTES were 0.68, 1.02, and 0.77 e, respectively. The binding energy results show that PAL (100) modification performance in the double-tooth state is the best compared to the other states, with the lowest value of −181.91 kJ/mol. The double-toothed state has lower barrier energy (94.69, 63.11, and 153.67 kJ/mol) during the modification process. This study offers theoretical insights into the chemical modification of the PAL (100) surface using APTES coupling agents, and can provide a guide for practical applications.

## 1. Introduction

**Figure 1:**
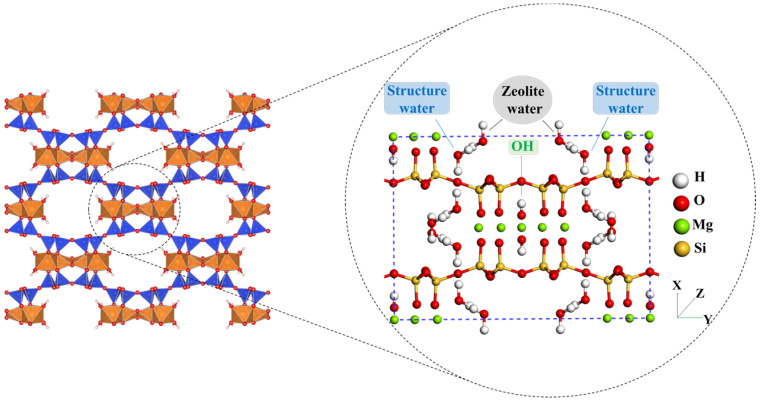
The microporous structure of palygorskite. Colour code: blue: Si; orange: Mg; red: O; white: H. Images produced with VESTA. Reprinted/adapted with permission from Ref. [14]. 2008, Momma, K.; Izumi, F.

**Figure 2:**
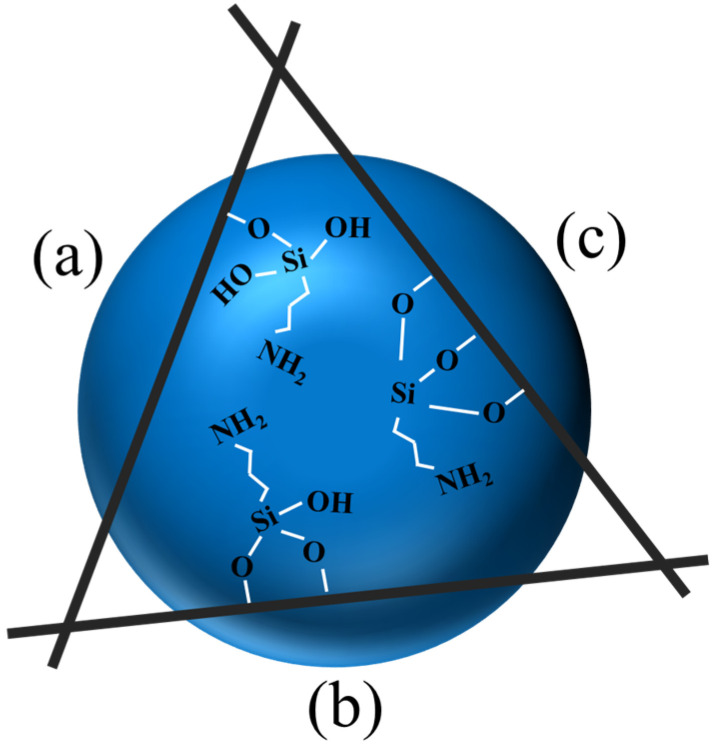
In the bonding reaction, coupling agents join in the (**a**) single-, (**b**) double-, and (**c**) three-toothed state.

Palygorskite (PAL) is a hydrous layered magnesium aluminum mineral of nanoscale fibrous architecture with the theoretical formula (MgAl)_2_Si_4_O_10_·4H_2_O. PAL can be described as a framework composed of inverted SiO_4_ tetrahedra linked by a chain of Si-O-Si bonds. The main structure of PAL is formed by these interwoven ribbons of 2:1 phyllosilicate structure. This structure is made up of two chains of Si-O tetrahedra and one Al-O octahedron. This arrangement defines the structural framework of PAL, shedding light on its distinct features and behaviour [1,2,3,4]. Due to its unique structure (see Figure 1), PAL has a large specific surface area, excellent stability, and good modification properties [5]. It has emerged as a highly promising adsorbent material, such as for the removal of dyes [6], gases [7], and hydrophobic pollutants [8] from the environment. The incorporation of iron (hydro) oxides into the pores and interlayers of PAL allows for the creation of optimal metallic sites which exhibit excellent arsenate retention capabilities [9]. Moreover, it has demonstrated remarkable performance in sequestering heavy metal contaminants during the remediation of polluted soils [10,11]. Its usage extends to the construction industry, where it serves as a thermal and acoustic insulation material. It is employed in the petrochemical, metallurgical, and nuclear industries as a filler and catalyst. Additionally, it serves as a filler in nanocomposites made of various polymers. However, their ability to disperse in polymeric matrices is hindered by surface polarity issues. Furthermore, PAL functionalization entails the addition of surface functional groups to this natural silicate, and these readily react with resin [12]. This functionalization enhances the interfacial connections between the resin and the PAL, resulting in higher thermal resistance of silicone adhesives derived from the material. These properties highlight the versatility and potential of PAL for various industrial and technological applications. Thus, PAL should be surface-modified to improve these interfacial interactions [13,14]. Grafting modifications can enhance material properties by improving surface functionality, enhancing stability, increasing chemical reactivity, and enabling better compatibility with other materials [15,16]. The coupling agent method is the most widely used method for surface modification of PAL. The chemical formula R-SiX_3_ can be used to describe the silane coupling agent. The first group, designated X, is a functional group that can hydrolyze, such as the -OCH_3_ or -OCH_2_CH_3_ groups, which can react with the -OH groups on PAL through dehydration and condensation. The second group, designated R, is an organic reactive group that does not hydrolyze, such as the C=C, -NH_2_, and -CH_2_Cl groups, among others, which can readily react with other organic groups and graft functional materials onto the surface. The 3-aminopropyltriethoxysilane (APTES) dilution solution and the silicon hydroxyl (Si-OH) groups can interact to form covalent bonds, which can subsequently graft onto the PAL surface [17]. Previously, many studies on the reaction mechanism and grafting effect of coupling agents have been reported in the literature. Wang et al. first used a silane coupling agent (APTES) to modify PAL nanorods and then used blending with epoxy epoxide to prepare nanocomposites. The authors believed that only one -OCH_2_CH_3_ group on the coupling agent participated in the reaction, as shown in Figure 2a [18]. Xue et al. reported the flip mechanism of the APTES molecule when it interacts with the PAL surface during the silylation process and stated that there are two -OCH_2_CH_3_ groups on the coupling agent that participate in the bonding reaction, as shown in Figure 2b [19,20]. Zhang et al. prepared polyvinylidene fluoride (PVDF)/PAL composite membranes that were grafted with the -NH_2_ group using the silane coupling agent APTES to enhance uniform dispersion in an organic polymer matrix. Moreira et al. synthesized APTES while modifying PAL for removal of the representative cationic and anionic dyes methylene blue and metanil yellow from aqueous solutions. In their research, they noted that the three -OCH_2_CH_3_ groups on the coupling agent were all involved in the bonding reaction, resulting in the structure of the product shown in Figure 2c [21,22]. The majority of past research on this subject, however, was only a small part of the synthesis. Although surface modification is important for PAL to function, it is unclear how the microscopic process influences macro-level performance given the level of research and testing techniques [23]. As a result, the reaction mechanism of the coupled-link modified PAL has to be examined with more precision.

In the area of materials science, computer simulations have revolutionized research by providing a powerful tool for understanding the intricate structure–performance relationships involved. These simulations allow for in-depth exploration of materials at the atomic and molecular levels, enabling valuable insights and efficient experimental design. In Zhang et al.’s study, molecular models of the interaction between the (100) crystal surface of a PAL coating and C_12_−C_18_ linear alkanes were built and subjected to molecular dynamics simulations in order to characterize the interactions between the base lubricant molecules and PAL coating [24]. Zhou et al. used molecular simulations to investigate the interaction between polylactic acid and PAL and predict their mechanical properties at different temperature [25]. In the present work, three types of APTES-PAL (100) resulting in single-, double-, and three-toothed states were developed to effectively determine the potential grafting reaction pathways via molecular simulations. This is a new pathway for the in-depth and detailed investigation of the chemical surface modification of PAL, providing a theoretical foundation for the chemical surface modification of PAL by coupling agents.

## 2. Results and Discussion

### 2.1. Surface Models

**Figure 3:**
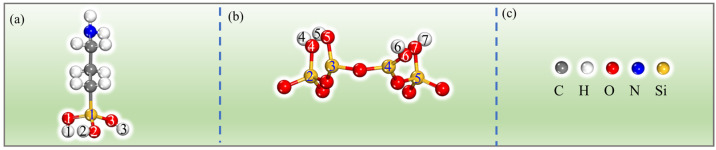
(**a**,**b**) represent the local structures of the PAL (100) surface and APTES, while (**c**) shows the color coding: the yellow, blue, red, and grey spheres represent Si, N, O, and C atoms, respectively.

**Figure 4:**
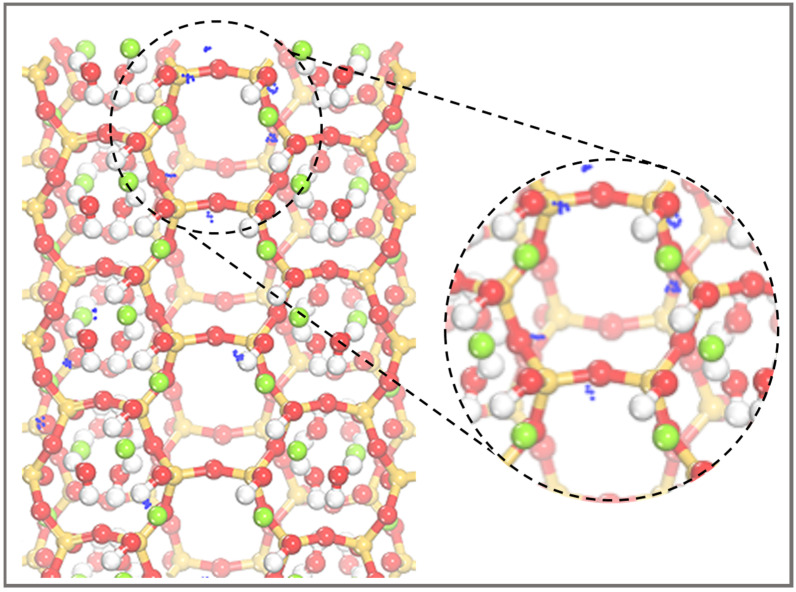
Active adsorption sites of APTES on the surface of PAL (100).

**Figure 5:**
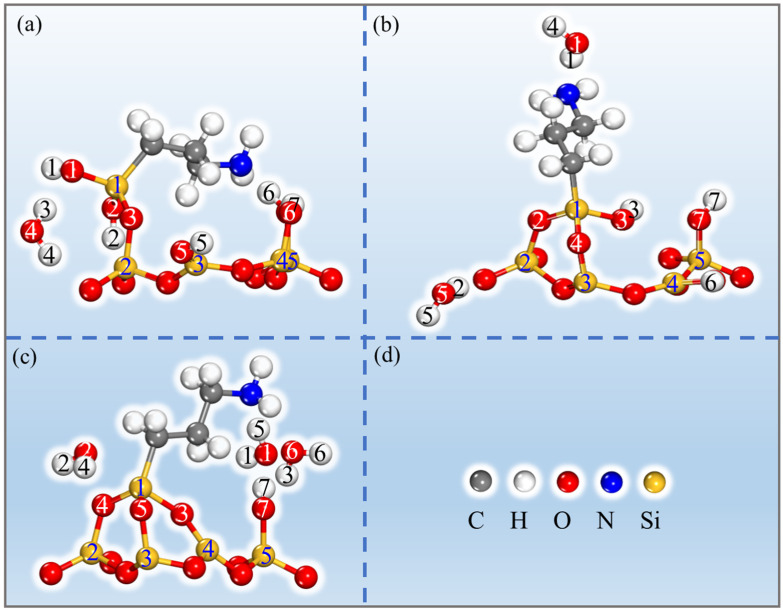
Configurations of APTES grafting on the surface of PAL (100) in the (**a**) single-toothed, (**b**) double-toothed, and (**c**) three-toothed state. The legend in (**d**) shows that the yellow, blue, red, and grey spheres represent Si, N, O, and C atoms, respectively.

When water interacts with a material, it can change its electrical characteristics and structural constitution [26,27]. Interfacial water can mediate interactions between modifiers and surfaces, which is crucial for the grafting of molecules onto the surface of the material. Previous studies have reported the presence of different forms of water in PAL as well as their limited mobility [24,28]. Additionally, the binding energies between alkanes and PAL have been found to be higher than alkane-water interactions [29]. Our research aims to provide a focused understanding of the grafting mechanism of APTES on the PAL surface without extensively exploring the influence of water molecules and the silane coupling agent. Considering our specific objectives, we simplified the model by not considering the interaction of water molecules with the silane coupling agent, allowing us to provide a more focused understanding of APTES grafting on the surface of PAL (100). To evaluate the impact of size effects in our model, which could cause flaws due to the system’s tiny size, we simplified the molecular model based on previous findings [7,30,31], specifically targeting the grafting of APTES onto the surface of PAL (100), with a focus on the Si-OH group in the SiO_4_ crystal structure. Our choice to simplify the PAL surface model by fixing the lattice parameters aimed to reduce computational complexity while preserving the essential surface features. Furthermore, we aligned our calculations with experimental procedures by acid treatment of the PAL prior to grafting. This alignment enhances the relevance and applicability of our findings, bridging the gap between theory and practice. In this research, the grafting of the APTES molecules on the surface of PAL (100) was studied; the optimized local geometric configurations of the isolated PAL (100) surface and APTES are shown in Figure 3. Figure 4 illustrates the active adsorption sites obtained through the Monte Carlo (MC) method. These active adsorption sites, depicted as blue spots, are predominantly located around the PAL surface. This indicates that the adsorption behavior of PAL is primarily influenced by the polar surface silanols, which exhibit strong interactions with APTES. To explain the surface modification mechanism of APTES on the surface of PAL (100), three theoretical models of APTES grafting on the surface were created, namely, the single-, double-, and three-toothed states. The calculation results for these configurations are displayed in Figure 5, and the corresponding bond lengths are summarized in Table 1. It is common knowledge that as the bond length decreases, the strength of the adhesive force between covalent bonds are inversely associated [32]. After DFT calculation, the APTES and PAL (100) surface were connected by chemical bonds (Si-O-Si) ranging from 1.587 to 1.802 Å, indicating strong surface interaction. This strong agreement with earlier experimental findings [17,33,34] demonstrates the validity of the simulation results.

### 2.2. The Performance of Surface Modification

The binding energies of all APTES-PAL (100) systems in the equilibrium state are listed in Table 1. From Table 1, it can be observed that the binding energies of the single-toothed and double-toothed states are negative, while the binding energy of the three-toothed state is positive. In addition, it can be seen that the smallest binding energy among all types of APTES-PAL (100) is that of the double-toothed state, which is −181.91 kJ/mol. Another important property in the case of grafting on the surface is the charge transfer. Charge transfer is a crucial phenomenon in grafting processes that involves the movement of electrons between the surface and the grafting agent. This electron transfer plays a significant role in determining the bonding strength and stability of the grafted molecules on the surface. A strong charge transfer enhances the attachment’s durability and stability while additionally influencing the electronic properties, reactivity, and functionality of the grafted surface. Considering charge transfer during grafting provides valuable insights into intermolecular interactions and aids in understanding and optimizing the grafting process [19,35]. Our calculation indicates that a partial electron transfer between APTES and the surface occurs, especially in the case of the double-toothed state showing the maximum electron transfer value of 1.02 e. This indicates that the double-toothed system is more stable and that the surface modification performance of this state is better than the others.

**Figure 6:**
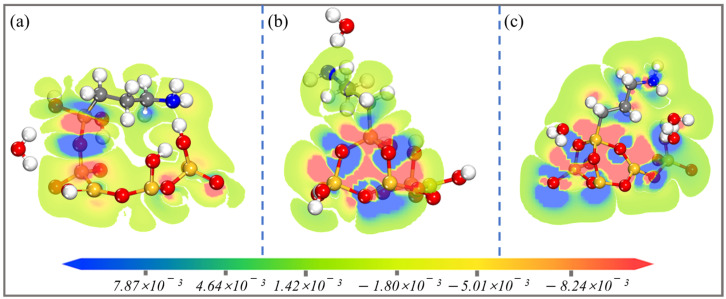
Electron density difference of the main Si-O-Si bonds in the (**a**) single-, (**b**) double-, and (**c**) three-toothed state APTES-PAL (100) models.

Electron density difference calculations were an integral part of our study, offering valuable insights into the redistribution of electrons upon the addition of APTES to the surface of PAL (100) [8]. By analyzing these electron density differences, we were able to gain a deeper understanding of the electronic interactions and charge transfer processes occurring within the system. This information is critical in elucidating the mechanism of APTES grafting and its impact on the surface properties of PAL, thereby paving the way for the design and development of novel functional materials with tailored electronic structures and enhanced performance. Figure 6 presents the electron density of APTES on the PAL (100) surface. The loss of electrons is shown by the red region, while the enrichment of electrons is shown by the blue region. There is a clear indication that for the single-toothed state the molecules on the surface of the PAL (100) experience charge loss. The areas where chemical bonds are formed are indicated by the blue regions. For the APTES molecules, charge redistribution occurs and the charge of the Si-O-Si bonds increases. These results indicate that APTES has strong adhesion to the surface of PAL (100).

### 2.3. Modification Mechanism

**Figure 7:**
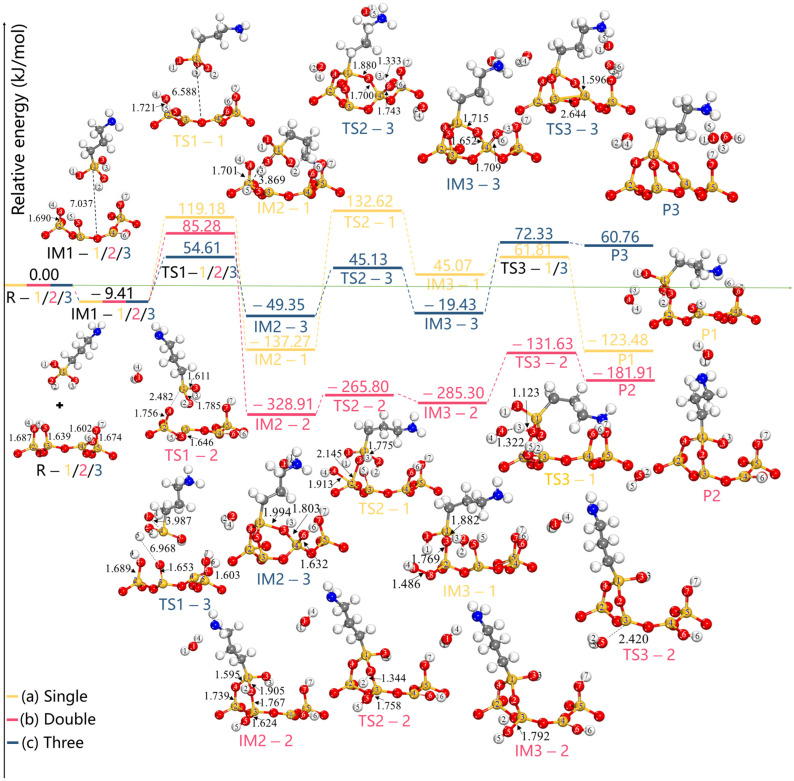
The potential energy profiles depict the modification path and optimized local configurations associated with the most probable grafting pathway of R, TS, and P for APTES grafting on the surface of PAL (100) in the (**a**) single-toothed, (**b**) double-toothed, and (**c**) three-toothed states. All bond distances are in Å.

**Figure 8:**
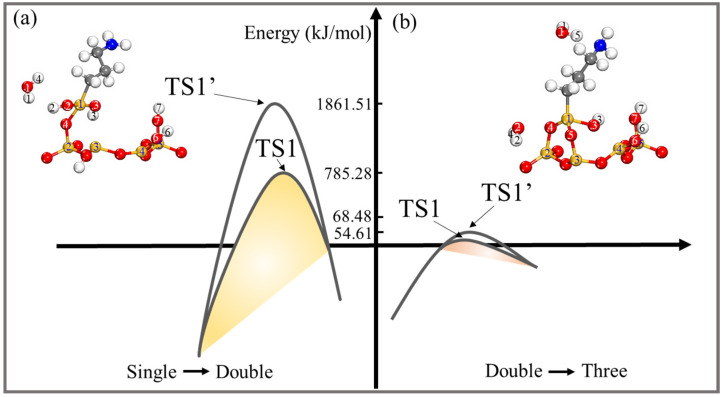
(**a**) Potential energy profiles for the path when using single-toothed APTES-PAL (100) to form double-toothed APTES-PAL (100) and (**b**) potential energy profiles for the path when using double-toothed APTES-PAL (100) to form three-toothed APTES-PAL (100). The different configurations of TS1 are plotted in Figure 7b,c, respectively. All bond distances are in Å.

By examining the potential energy profiles, it was possible to predict the thermodynamic properties of the pathways of the grafting reactions for the single-, double-, and three-toothed states of APTES-PAL (100) and thoroughly investigate the reaction configurations of the most advantageous pathway. Figure 7 and Table 1 show the obtained information on the most stable grafted configurations of the species in the major reaction path. The configurations of potential intermediates and products were explored using the computed modification routes of the PAL (100) surface, which can be used as a reference for modelling the reactants (R), intermediates (IM), and products (P) to precisely locate the transition state (TS). The direction of the grafting reactions is frequently dominated by trigger bonds [36,37]. Si^2^(PAL)-O^4^(PAL), Si^4^(PAL)-O^1^(APTES), and Si^4^(PAL)-O^6^(PAL) bonds can be regarded as trigger bonds for the single-, double-, and three-toothed states of the APTES-PAL (100) molecule, respectively. Initially, for all three grafted states the APTES moves toward the PAL (100) surface to reach IM1. Then, as shown in Figure 7a, Si^2^(PAL) attracts O^3^(APTES) with a distance of 3.869 Å; the activation energy of TS1 is 119.18 kJ/mol. In IM3, the O^3^(APTES) and Si^2^(PAL) atoms form a bond and the O^4^(PAL)-Si^2^(PAL) starts to break. Meanwhile, a new bond is formed between the O^8^(PAL) and Si^2^(PAL) atoms. The distances of O^3^(APTES)-Si^2^(PAL) and O^8^(PAL)-Si^2^(PAL) are 1.769 and 1.486 Å, respectively. The activation energy of this step is 132.62 kJ/mol. Then, the H^4^(PAL)-O^4^(PAL) bond breaks off the H^4^(PAL)-O^4^(PAL)-Si^2^(PAL) bond and the H^4^(PAL)-O^4^(PAL) moves toward H^3^(APTES) atom through TS3, where both the Si^1^(APTES) and Si^2^(PAL) atoms link with the O^3^(APTES) atom with bond lengths of 1.679 and 1.670 Å, respectively. This process is exothermic by *−*123.48 kJ/mol, with an activation barrier of 61.81 kJ/mol. Figure 7b indicates that the O^1^(APTES)-Si^1^(APTES) and H^2^(APTES)-O^4^(PAL) bonds are broken to form H_2_O when the APTES gets close enough to the PAL (100) surface to reach TS1. The other atoms of the APTES transfer to the PAL (100) surface, forming O^4^(PAL)-Si^1^(APTES) (1.595 Å) and O^2^(APTES)-Si^3^(PAL) (1.767 Å) bonds, whereas the remaining O^5^(PAL)-H^5^(PAL) continues to be bound to the Si^3^(PAL) atom with a shorter bond length of 1.624 Å. As the reaction proceeds, H^2^(APTES) breaks off from the O^2^(APTES)-H^2^(APTES) bond and joins O^5^(PAL) to form an O^5^(PAL)-H^2^(APTES) bond, then, the O^5^(PAL)-Si^3^(PAL) bond starts to break and the new H_2_O molecule moves away from the PAL (100) surface. The activation energy for the products is found to be *−*131.63 kJ/mol, which indicates that this process can be performed at room temperature. From Figure 7c, it can be seen that the activated H^5^(PAL) atom shifts to the O^1^(APTES)-H^1^(APTES), forming an H_2_O molecule, while the O^2^(APTES)-H^2^(APTES) moves to the PAL of the O^4^(PAL) atom, forming another H_2_O molecule. At the same time, the O^4^(PAL) and O^5^(PAL) atoms combine with the Si^1^(APTES) atom to produce O^4^(PAL)-Si^1^(APTES) and O^5^(PAL)-Si^1^(APTES) bonds; the O^3^(APTES) atom tends to bind to Si^4^(PAL), resulting in a longer O^3^(APTES)-Si^4^(PAL) bond (1.803 Å). Sequentially, O^3^(APTES) captures the Si^6^(PAL) connected to the O^3^(APTES)-Si^6^(PAL) bond. This step proceeds via TS2, in which the O^6^(PAL)-Si^4^(PAL) bond is stretched to 1.709 Å. Finally, the O^6^(PAL)-Si^4^(PAL) bond ruptured and releases H_2_O. This step has a very large activation barrier of 72.33 kJ/mol and is largely endothermic by 60.76 kJ/mol. The double-toothed state APTES-PAL (100) exhibits the most favorable energetics. This observation aligns with the conditions used in the different grafting reactions of APTES-PAL in the single-, double-, and three-toothed states in experimental studies [18,19,22]. Wang et al. prepared single-toothed APTES-PAL using magnetic stirring and refluxing at 80 °C [18]. Xue et al. achieved double-toothed APTES-PAL by introducing terminal amino groups onto the PAL surface at 75 °C [19]. Similarly, Moreira et al. performed PAL functionalization with APTES at 80 °C in both dry and aqueous solvents to obtain three-toothed APTES-PAL [22]. These studies collectively demonstrate the relative simplicity of constructing APTES-PAL in the double-toothed state. Our simulations support these findings, as we show that double-toothed APTES-PAL (100) exhibits the most favorable energetics. This consistency between our results and the experimental conditions for grafting reactions validates the reliability of our simulations. Consequently, our simulations provide confirmation of the reliability of the present findings. The consistency between our results and the experimental conditions for grafting reactions further strengthens the validity of our simulations. Furthermore, we conducted investigations into how the single- and double-toothed states of APTES-PAL (100) give rise to the double- and three-toothed states, respectively. In our work, only the initial transition state is discussed, and is set as TS1′ in Figure 8a,b. The activation energies reaching TS1′ were obtained as 1861.51 and 68.48 kJ/mol, respectively, which are both higher than the isolated APTES and PAL (100) surface grafting. This indicates that it is more difficult to perform this process.

### 2.4. Electronic Structures

**Figure 9:**
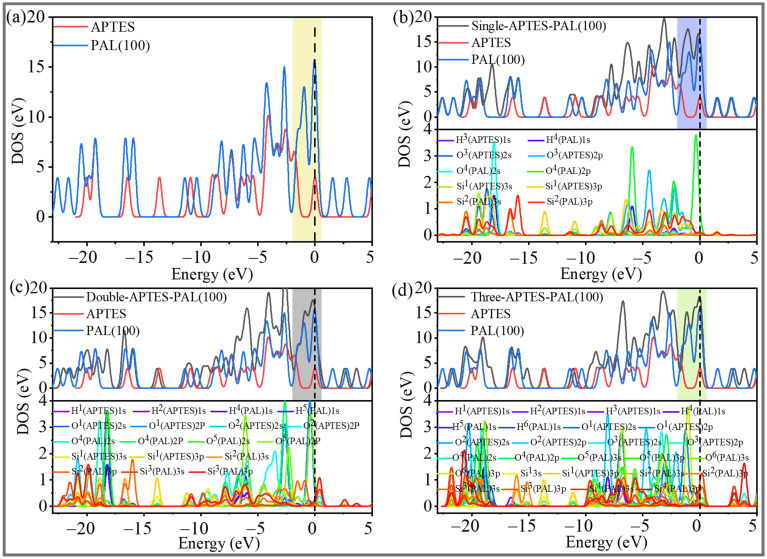
The TDOS and PDOS of (**a**) the individual APTES and the PAL (100) surface and (**b**) the single-toothed, (**c**) double-toothed, and (**d**) three−toothed state APTES-PAL (100) surfaces. The broken vertical line represents the Fermi energy.

The computations for the total densities of states (TDOS) and partial densities of states (PDOS) were carried out for the APTES-grafted PAL (100) surface, as detailed in Figure 9. A high TDOS value intensity near the Fermi level, which denotes a high overall system energy and has been extensively studied in previous work, leads to an unstable state [38,39,40]. The TDOS peak of the double-toothed APTES-PAL (100) near the Fermi level is lower than that of the other states in Figure 9a–d. This indicates that the most stable structure is the double-toothed state and that the stability sequence is double-toothed state > single-toothed state > three-toothed state, which is in good agreement with the binding energy and charge transfer results shown in Table 1. The orbital hybridization is undoubtedly advantageous to the creation of a stable grafted structure, according to the PDOS of a number of key atoms. As shown in Figure 9b, in PDOS we found that O^3^(APTES)-Si^2^(PAL) interaction is realised by the hybridization of the 2p orbital of O^3^(APTES) with the 3s and 3p orbitals of Si^2^(PAL), with the hybridization region being closer to the Fermi energy level. From *−*20 to *−*17 eV and *−*7 to *−*2 eV, the large overlapping area between H^3^(APTES) 1s, H^4^(PAL) 1s, and O^4^(PAL) 2s, 2p indicates that their orbits are highly hybridized during the modification process. In Figure 9c, the increase of PDOS from *−*10 to *−*1 eV is mainly contributed by the 2s and 2p orbitals of O^1^(APTES) and O^5^(PAL) and the 1s orbital of the H^1^(APTES), H^2^(APTES), H^4^(PAL), and H^5^(PAL) atoms. There are obvious overlaps with the 1s orbitals of the H^1^(APTES), H^4^(PAL), and H^2^(APTES), H^5^(PAL) atoms near *−*7 to *−*5 eV, respectively. As they overlap, there is electrical interaction between them, and chemical connections bind them together. The hybridization between the 3s and 3p orbitals of Si^1^(APTES), Si^2^(PAL), Si^3^(PAL), and the 2p orbital of O^2^(APTES) and O^4^(PAL) can be found from *−*10 to *−*1 eV, indicating the bonding states. A similar situation can be observed for the three-toothed state APTES-PAL (100), as plotted in Figure 9d. In the range of *−*10 ~ *−*5 eV, the O^4^(PAL) atom has a strong electron orbital interaction with the Si^1^(APTES) and Si^2^(PAL) atoms, and they are joined by a chemical bond. The Si^1^(APTES)-O^5^(PAL)-Si^3^(PAL) hybrid and Si^1^(APTES)-O^3^(APTES)-Si^4^(PAL) hybrid are similar to Si1(APTES)-O4(PAL)-Si^2^(PAL). In addition, the hybridization between H^2^(APTES), H^4^(PAL) 1s states and O^2^(APTES) 2p states occurs around the energy level of *−*8 to *−*3 eV. The splitting and broadening of peaks indicate a strong interaction and bond formation. While H^1^(APTES)-O^1^(APTES)-H^5^(PAL) and H^3^(APTES)-O^6^(PAL)-H^6^(PAL) hybrid energy level can be found ranging from *−*19 to *−*17 eV and *−*7 to *−*4 eV, bonding states exist as well.

## 3. Computational Details

The primary reactivity of PAL with silane coupling agents occurs through the formation of Si-O-Si bonds, as demonstrated by Yang et al. [41,42]. Furthermore, Zhu et al. reported that acid treatment of PAL results in the removal of metal cations, exposing abundant active sites for improved grafting interactions with APTES [43,44]. Experimental observations, including the broadening and weakening of the Si-O-Si stretching band at 1195 cm^−1^ and the disappearance of bands at 3614, 3550, and 984 cm^−1^ in the infrared spectra, support these findings. These bands were initially assigned to the asymmetric stretching of Mg-OH, antisymmetric stretching of Al-Fe-OH (Al-Mg-OH band), and asymmetric stretching of perpendicular Si-nonbridging oxygen-Mg (Si-O-Mg), respectively, indicating the partial removal of Mg^2+^ and Al^3+^ ions [45]. Consequently, it can be inferred that the primary reactivity of PAL with APTES occurs in the Si-OH groups. Our analysis of the active sites for APTES on the acid-treated PAL surface is consistent with experimental results, confirming the preferential distribution of APTES around Si-OH groups (see Figure 4). To ensure computational efficiency and model reliability, we drew inspiration from the DFT calculations conducted by Peng et al. on the composite of tetramethylguanidine/PAL and Liu et al. on the interaction between polyethylene glycol and PAL [46,47]. Additionally, we followed the methodology introduced by Karamanis et al. which employed a primitive rhombohedral cell (one quarter of the conventional cell) to reduce the computational complexity associated with studying the siliceous structure of faujasite [48]. In contrast to their work, we simplified the model by focusing on the PAL (100) surface, specifically the Si-OH groups, the SiO_4_ units connected to them, and the Si-O-Si framework linked to AlO_8_. These simplifications allowed us to strike a balance between computational feasibility and maintaining the accuracy and reliability of the model while ensuring logical consistency throughout the study [49,50,51,52]. Initially, the crystal structure of PAL was optimized to ensure stability. Subsequently, the PAL (100) surface was built by (100) cleaving of the crystal model, which has previously been used to successfully simulate PAL [24,25,47]. Then, the cell was expanded to a 2 × 2 × 2 configuration and relaxed to achieve a stable structure. Following this, the lattice parameters of the surface model were were fixed and the SiOH groups within the SiO_4_ and AlO_8_ frameworks, which serve as the active sites, were isolated and subjected to optimization for further examination of the mechanism involving APTES. In order to understand the interactions between atoms and molecules, molecular simulation studies were adopted using the DMol^3^ programme in the modeling software Materials Studio 8.0 [53]. All computations used the generalized gradient approximation with the Perdew-Burke-Ernzerhof (PBE) exchange correlation functional [54]. A 1 ×1 × 1 Monkhorst–Pack k-point sampling with a Methfessel–Paxton smearing of 0.005 Ha was employed [55]. The double numerical plus polarization (DNP) basis set was employed to expand the electronic eigenstates, and all core and valence electrons were taken into explicit consideration. Calculations included determining the APTES-PAL (100) system’s electronic structure as well as its equilibrium geometries, vibrational frequencies, and energetics. Frequency calculations were performed after the optimisations to ensure that the optimised structures were all minimal structures (all >0 frequencies). Different grafted configurations of the coupling agents over the hydrogenated palygorskite surfaces were investigated. However, only the most stable configurations are reported here. The Adsorption Locator module utilized the MC method. In this simulation, the optimized structures of APTES were considered as adsorbates. A maximum adsorption distance of 6 Å was applied. To calculate the binding energy (*E*_bind_) and evaluate the strength of the interaction between the surface and the silane coupling agents, the following Formula (1) was used [56,57]:

*E*_bind_ = *E*_total_ − (*E*_PAL(100)_ + *E*_APTES_) (1)

where *E*_PAL(100)_ and *E*_APTES_ are the single-point energies of the individual PAL and APTES molecules, respectively and *E*_bind_ < 0 denotes an attractive rather than a repulsive interaction force between the APTES molecules and the (100) plane of PAL, with a higher attracting force indicated by a lower binding energy.

To learn more about the mechanism by which APTES interacts with the surface of PAL (100), the charge transfer *Q*_t_ was calculated via Hirshfeld analysis. The *Q*_t_ in the grafting process was obtained by Formula (2):

*Q*_t_ = *Q*_grafted_ − *Q*_iso_ (2)

where the charges of the grafted and isolated PAL (100) surface are *Q*_grafted_ and *Q*_iso_, respectively. If the Hirshfeld charge (e) value is more than zero the atoms are negatively charged, while if it is less than zero, they are positively charged.

,The transition states (TS) were searched using the comprehensive linear synchronous transit (LST) or quadratic synchronous transit (QST) approach. After performing linear synchronous transit (LST) maximization, energy was minimized in directions that were conjugated to the reaction pathway [58]. The quadratic synchronous transit (QST) maximizing process was carried out using the TS approximation generated in this manner. Another minimization of a conjugate gradient was then performed. The same method was repeated until a stationary spot was located. The 0.25 eV/Å per-atom convergence threshold for the TS search was determined to be the root-mean-square force [59].

## 4. Conclusions

In summary, this study investigated the modification mechanism of APTES on the surface of PAL (100) using first-principles simulations. The results reveal strong grafting of APTES molecules at the -OH groups of the PAL (100) surface in the single-, double-, and three-toothed states with binding energies of −123.48, −181.91, and 60.76 kJ/mol, respectively. Significant charge transfers are observed during the grafting process, with the double-toothed state exhibiting the highest charge transfer of 1.02 e from the surface to APTES. The DOS projections show peak shifts, indicating strengthened interaction through chemical bonds. The interaction between APTES and the PAL (100) surface involves overlapping of the outer orbitals between O and Si or H atoms. Furthermore, the energy barrier for the double-toothed state is lower than that of the single-toothed and three-toothed states, suggesting that grafting APTES on the PAL (100) surface through two Si-O-Si bonds is energetically favored. Overall, the double-toothed state of APTES-PAL (100) is the most energetically advantageous.

## Figures and Tables

**Table 1:** Energies and geometric parameters of the most stable grafted product configurations.

Configurations	*E*_bind_ (kJ/mol)	Total Charge Transfer *Q*_t_ (e)	Bond Type	Length (Å)
Single	−123.48	0.68	Si^1^(APTES)-O^3^(APTES)	1.679
			O^3^(APTES)-Si^2^(PAL)	1.670
Double	−181.91	1.02	Si^1^(APTES)-O^4^(PAL)	1.632
			Si^1^(APTES)-O^2^(APTES)	1.723
			O^4^(PAL)-Si^2^(PAL)	1.715
			O^2^(APTES)-Si^3^(PAL)	1.622
Three	60.76	0.77	Si^1^(APTES)-O^3^(PAL)	1.802
			Si^1^(APTES)-O^4^(PAL)	1.662
			Si^1^(APTES)-O^5^(PAL)	1.690
			O^3^(APTES)-Si^4^(PAL)	1.587
			O^4^(PAL)-Si^2^(PAL)	1.683
			O^5^(PAL)-Si^3^(PAL)	1.675

## Data Availability

The materials and datasets generated and analyzed during the current study are publicly available from the corresponding author on reasonable request.

## Sample Availability

Samples of the compounds are available from the authors.
